# A pooled analysis of mesenchymal stem cell-based therapy for liver disease

**DOI:** 10.1186/s13287-018-0816-2

**Published:** 2018-03-21

**Authors:** Lu Zhao, Shanquan Chen, Xiaowei Shi, Hongcui Cao, Lanjuan Li

**Affiliations:** 1 State Key Laboratory for Diagnosis and Treatment of Infectious Diseases, the First Affiliated Hospital, College of Medicine, Zhejiang University, Collaborative Innovation Center for Diagnosis and Treatment of Infectious Diseases, 79 Qingchun Rd, Hangzhou City, 310003 China; 2 The Jockey Club School of Public Health and Primary Care, The Chinese University of Hong Kong, Hong Kong, 999077 China; 3 Chu Kochen Honors College, Zhejiang University, 866 Yuhangtang Rd, Hangzhou City, 310058 China

**Keywords:** Pooled analysis, Mesenchymal stem cell, Cell therapy, Liver disease

## Abstract

**Background:**

Liver disease is a major cause of death and disability. Mesenchymal stem cells (MSCs) show promise for the treatment of liver disease. However, whether MSC-based therapy is more effective than conventional treatment is unclear, as are the optimal MSC source, the administration frequency, and the most effective MSC delivery route. We therefore undertook a systematic review and meta-analysis of the therapeutic efficacy of MSCs against liver disease and the related factors.

**Methods:**

We systematically searched Medline (PubMed), Cochrane Library, EMBASE, ClinicalTrials.gov, and SinoMed CBM to identify studies published up to June 2017 involving liver disease patients receiving MSC-based therapy and which reported estimates of liver function during the follow-up period.

**Results:**

Thirty-nine studies were selected from 672 publications. According to a meta-analysis of 23 controlled studies, compared with conventional treatment MSC therapy significantly improves liver function in patients with liver disease in terms of the model of end-stage liver disease score, albumin, alanine aminotransferase, and total bilirubin levels, and prothrombin time, up to 6 months after administration. However, it has no beneficial effects in terms of prothrombin activity, international normalized ratio, or cholinesterase level. Considerable heterogeneity was identified at most time points. Subgroup analyses showed that a single MSC injection was more effective than multiple injections, MSC administration was more effective via the hepatic artery than the peripheral vein, and MSCs derived from bone marrow were more effective than those derived from the umbilical cord.

**Conclusions:**

MSC-based therapy is relatively safe and improves liver function during the first 6 months after administration. A single injection administration via the hepatic artery and MSCs derived from bone marrow are optimal in terms of improving liver function. However the significant heterogeneity among studies and discontinuous results of the subgroup meta-analysis should be addressed; moreover the long-term efficacy of MSC therapy warrants further investigation.

**Electronic supplementary material:**

The online version of this article (10.1186/s13287-018-0816-2) contains supplementary material, which is available to authorized users.

## Background

Liver disease, including viral hepatitis, alcoholic liver disease, nonalcoholic fatty liver disease, and associated end-stage liver disease, is a global health concern. The incidence of liver disease is projected to increase because of new cases of hepatitis [1], the increasing prevalence of obesity and lack of physical activity [2], and alcohol consumption [3]. For example, chronic hepatitis, the most common cause of end-stage liver disease [2], affects approximately 325 million people and caused 1.34 million deaths globally in 2015, and this number is increasing [1].

In general, without efficient treatment, all types of chronic hepatitis will progress to end-stage liver disease, such as cirrhosis, chronic liver failure, and hepatocellular carcinoma [2], which has a poor long-term clinical outcome. There has been a substantial increase in the burden of cirrhosis and other chronic liver diseases; indeed, the numbers of disability-adjusted life years (from 31 to 39 million) and deaths (from 0.9 to 1.3 million) increased from 1990 to 2015 [4]. In 2015, approximately 788,000 people died due to hepatocellular carcinoma, the second leading cause of cancer-related death worldwide [5].

When chronic hepatitis progresses to end-stage liver disease, conventional management for liver failure generally does little to promote hepatic repair despite the availability of a broad array of treatment options. Currently, the only curative treatment for end-stage liver disease is liver transplantation, but donor shortage and waiting list mortality, high costs, long-term side effects, and postoperative morbidity and mortality severely limit its application [6, 7].

Consequently, the quest for novel therapeutic options has resulted in the emergence of growth factor-, gene-, probiotic-, and cell-based therapies. Cell-based therapy using mesenchymal stem cells (MSCs) shows considerable promise [8]. MSCs are plate-adhering, fibroblast-like cells with self-renewal capacity and the ability to differentiate into multiple mesenchymal cell lineages, such as adipocytes and chondrocytes. First proposed in the 1980s by Arnold Caplan [9], MSCs and MSC-based therapy have been the subject of *in vitro*, *in vivo*, and clinical studies for the treatment of liver diseases. However, whether MSC-based therapy is more effective than conventional treatment against liver disease is unclear since studies have reported a greater [10] or similar [11] efficacy compared with conventional treatment, and one study [12] reported that MSCs exert a deleterious effect on the liver. Two meta-analyses assessed the effect of MSCs on hepatic repair, but the findings were inconsistent [13, 14]. Several factors must be considered when evaluating the efficacy of MSC-based therapy against liver disease. For example, the MSC subpopulation used is related to the therapeutic efficacy [15]. Other factors such as the cell type, delivery route, and single or multiple injections may also influence the efficacy of MSC therapy. However, these factors were not considered in previous meta-analyses [13, 14]. We therefore undertook a systematic review and meta-analysis of the therapeutic efficacy of MSCs against liver disease and the factors involved.

## Methods

### Search strategy and selection criteria

We searched Medline (PubMed) [16], Cochrane Library [17], EMBASE [18], ClinicalTrials.gov [19], and SinoMed CBM [20] (up to June 2017) to identify relevant studies using a combined free text and MeSH heading search strategy (see Additional file 1), with no language or time restrictions. The retrieval strategy was conducted based on the patient–intervention–comparison–outcome principle and comprised keywords related to liver disease (*e.g.*, “liver”, “liver disease*”, “hepatitis”, “hepatic fibrosis”, “liver fibrosis”, “liver cirrhosis”, “liver neoplasm*”, “liver failure”, “fatty liver”, “liver abscess”, and “liver injury”) and mesenchymal stem cells (“mesenchymal stem cell*”, “mesenchymal stromal cell*”, and “multipotent stromal cell*”). The reference lists of the retrieved studies were also checked for relevant studies. The inclusion criteria were: 1) clinical studies; 2) MSCs; 3) patients diagnosed with liver disease; and 4) availability of liver function parameters (model for end-stage liver disease (MELD) score; levels of albumin (ALB), alanine aminotransferase (ALT), total bilirubin (TBiL), and cholinesterase (CHE); prothrombin time (PT); prothrombin activity (PTA); and international normalized ratio (INR)). Studies were excluded if they were animal-based, reviews, or case reports, if the full text was not available, or if the study information was inadequate. When duplicate reports from the same study were identified, only the one with more information or the longer follow-up period was included.

### Data extraction and statistical analysis

For each study, data were extracted by one investigator and checked by a second investigator to ensure accuracy. Information on the following was extracted: patients (number, age, sex, and liver disease type), MSCs (number, type, delivery route, and frequency of administration), liver function parameters during the follow-up period, and study information (author, publication year, country, study design, and follow-up period).

A meta-analysis of 23 controlled trials was conducted to evaluate whether the efficacy of MSC-based therapy was greater than that of conventional therapy in terms of liver function improvement. Liver function parameters were evaluated by calculating the standardized mean difference (SMD) with 95% confidence interval (CI). The percentage of variability across studies that was attributable to heterogeneity beyond chance was assessed using the chi squared-based *Q* test (*P* < 0.1 was considered indicative of significance) and *I*^2^ statistic (*I*^2^ > 50% indicated high heterogeneity). A Forest plot was used to visualize the SMD and 95% CI for each study. A random effects model was used since this method provides a more conservative estimate of the presence of heterogeneity. A sensitivity analysis with omission of one study at a time was conducted to identify heterogeneity. Where sufficient studies were available, publication bias was assessed by the Egger test and visualized using Begg funnel plots [21]. Contour-enhanced funnel plots [22] were generated, and a sensitivity analysis using the trim and fill method [23] was conducted to explore publication bias if a plot revealed asymmetry. Subgroup meta-analyses were conducted to identify factors related to the therapeutic efficacy of MSCs; the factors investigated were cell source (bone marrow or umbilical cord), MSC delivery route (peripheral vein or hepatic artery), MSC administration frequency (single or multiple injections), and study design (randomized controlled trial (RCT) or not (nRCT)). All analyses were conducted using R (version 3.4.0) and SPSS (version 21) software.

## Results

### Study selection

**Fig. 1:**
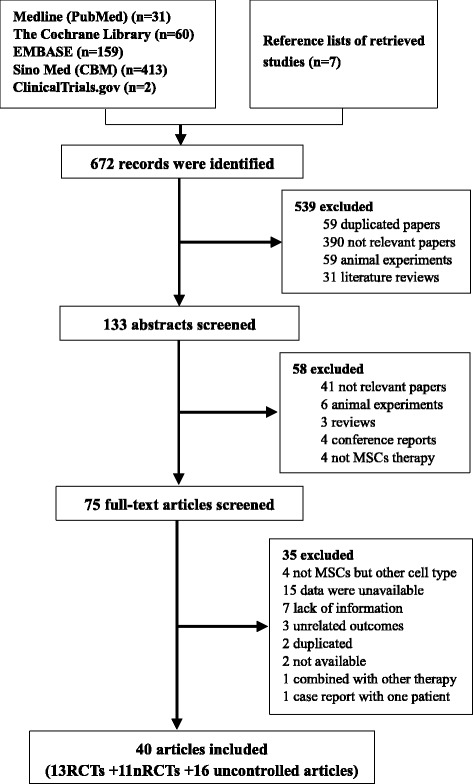
Flowchart of study selection. MSC mesenchymal stem cell, nRCT, nonrandomized controlled trial, RCT randomized controlled trial

As shown in Fig. 1, a total of 672 potentially eligible articles were identified by searching the five databases and the reference lists of the retrieved studies. Of these, 59 duplicated articles were excluded, along with 480 articles deemed irrelevant after reading the title and 58 articles after reading the abstract. After assessment of the full texts, 35 articles were excluded based on the exclusion criteria. Two papers [24, 25] based on the same study were included because they reported different outcome parameters. In total, 39 studies (40 articles) with 24 controlled trials (13 RCTs and 11 nRCTs) and 15 uncontrolled trials were ultimately identified as relevant. Of the 11 nRCTs, one [26] was regarded as an uncontrolled trial because the control group data could not be extracted. Thus, 13 RCTs [11, 24, 25, 27–37] (*n* = 624) and 10 nRCTs [38–47] (*n* = 525) involving 592 patients in the MSC group (received MSC-based therapy) and 557 patients in the control group (received conventional supportive treatment), as well as 16 uncontrolled trials [26, 48–62] (*n* = 391) were analyzed.

### Characteristics of the included studies

The characteristics of the 39 studies are presented in Table 1. The studies were published between 2010 and 2017 and included a total of 1540 patients with an average age of 46.7 years, and were conducted in China, Egypt, Iran, Turkey, and Korea. The sample size ranged from 7 to 110 participants. The MSCs were derived from the bone marrow (B-MSCs; *n* = 19 studies) or umbilical cord (UC-MSCs; *n* = 20 studies) and were administered via the peripheral vein (*n* = 20), hepatic artery (*n* = 13), portal vein (*n* = 1), or multiple routes (*n* = 5). A single MSC injection was performed in 23 studies and multiple MSC injections in 16 studies. The follow-up period ranged from 1 to 36 months. The studies included patients with decompensated or advanced liver cirrhosis (*n* = 19), liver cirrhosis (*n* = 7), acute-on-chronic, chronic, or end-stage liver failure (*n* = 10), liver fibrosis (*n* = 1), autoimmune liver disease (*n* = 1), and end-stage liver disease (*n* = 1).

**Table 1:** Character of included studies

Study	Country	Study design	Patient populations	Sample size (MSC/control)	Average age (MSC/control)	Male (%)	Cell type	Delivery route	Times of injection	Dosage of MSCs/ml	Number of MSCs	Maximum follow-up (months)
Y. Du, 2011 [37]	China	nRCT	Decompensated liver cirrhosis	25/25	53	82.0%	UC-MSCs	Artery	Multiple	1	(0.1–1.0) × 10^8^ cells	2
L. Peng, 2011 [38]	China	nRCT	Liver failure	6/15	42.19/42.22	94.3%	B-MSCs	Artery	Single	10	1 × 10^7^ cells	12
Amer ME, 2011 [27]	Egypt	RCT	End-stage liver failure	20/20	50.5/50.0	82.5%	B-MSCs	Intrasplenic vs intrahepatic	Single	5	NR	6
H. Lin, 2012 [24, 25]	China	RCT	Decompensated liver cirrhosis	38/16	47/48	90.7%	UC-MSCs	Vein	Multiple	60	(0.5–1.0) × 10^6^/kg	12
Z. Zhang, 2012 [28]	China	RCT	Decompensated liver cirrhosis	30/15	48/47	88.9%	UC-MSCs	Vein	Multiple	NR	0.5 × 10^6^/kg	12
El-Ansary M, 2012 [39]	Egypt	nRCT	Advanced liver cirrhosis	15/10	48/51.6	76.0%	B-MSCs	Vein	Single	NR	1 × 10^6^/kg	6
J. Yu, 2012 [40]	China	nRCT	End-stage liver failure	13/22	43.46/47.45	82.9%	B-MSCs	Vein	Single	100	5 × 10^6^ cells	1
Y. Zhang, 2012 [29]	China	RCT	Decompensated liver cirrhosis	12/18	48.63/49.86	70.0%	UC-MSCs	Artery	Single	10	≥ 2 × 10^7^ cells	3
S. Ouyang, 2013 [41]	China	nRCT	Decompensated liver cirrhosis	33/34	53.1/55.2	83.6%	B-MSCs	Artery	Single	5	(3–5) × 10^6^ cells	6
M. Zhu, 2013[42]	China	nRCT	Decompensated liver cirrhosis	22/20	48.5/49.2	81.0%	UC-MSCs	Vein	Multiple	100	1 × 10^8^ cells	2
B. Liu, 2013[43]	China	nRCT	Acute-on-chronic liver failure	16/19	38.7	80.0%	UC-MSCs	Vein vs artery	Single	NR	> 5 × 10^7^ cells	3
Q. Wang, 2013 [30]	China	RCT	Chronic liver failure	9/9	50.71 (median)	85.7%	UC-MSCs	Vein	Multiple	NR	(1.2–6.2) × 10^7^/ml	4
L. Xu, 2014 [31]	China	RCT	Liver cirrhosis	20/19	44/45	61.5%	B-MSCs	Artery	Single	20	4 × 10^7^ cells	6
Mohamadnejad M, 2013 [11]	Iran	RCT	Decompensated liver cirrhosis	14/11	43.1/34.6	52.0%	B-MSCs	Vein	Single	100	1.95 × 10^8^ cells	12
H. Salama, 2014 [32]	Egypt	RCT	End-stage liver disease	20/20	50.27/50.9	82.5%	B-MSCs	Vein	Single	100	1 × 10^6^/kg	6
L. Zhuang, 2014 [44]	China	nRCT	Liver cirrhosis	29/35	55/56	78.1%	UC-MSCs	Portal vein	Single	NR	0.5 × 10^8^ cells	6
H. He, 2015 [45]	China	nRCT	Acute-on-chronic liver failure	46/54	43.8/44.5	89.0%	UC-MSCs	Vein	Multiple	100	1 × 10^5^/kg	3
H. Luo, 2015 [46]	China	nRCT	Decompensated liver cirrhosis	50/46	43/44	88.5%	UC-MSCs	Vein	Multiple	100	NR	3
L. Tong, 2015 [47]	China	nRCT	Decompensated liver cirrhosis	20/20	56.4/56	55.0%	UC-MSCs	Vein	Multiple	100	(2–4) × 10^9^ cells	6
Y. Li, 2015 [33]	China	RCT	Acute-on-chronic liver failure	31/27	41.6/43.1	89.7%	UC-MSCs	Vein	Multiple	60	(0.5–1.0) × 10^6^/kg	12
D. Zhang, 2016 [36]	China	RCT	Hepatic fibrosis	30/30	30.98/32.1	55.0%	B-MSCs	Vein	Multiple	2	6 × l0^6^ cells	3
K.T. Suk, 2017 [34]	South Korea	RCT	Liver cirrhosis	37/18	53.7/53.7	89.1%	B-MSCs	Artery	Single vs multiple	10	5 × 10^7^ cells	12
B.-L. Lin, 2017 [35]	China	RCT	Acute-on-chronic liver failure	56/54	40/42.8	94.5%	B-MSCs	Vein	Multiple	10	1 × 10^3^ to 1 × 10^5^/kg	6
J. Shen, 2015 [26]^a^	China	nRCT	Decompensated liver cirrhosis	28/28	42.4	94.6%	B-MSCs	Artery	Single	NR	(1.7 ± 0.4) × 10^7^/ml	36
Kantarcioʇlu M, 2015 [62]	Turkey	Uncontrolled	Liver cirrhosis	12	39	58.3%	B-MSCs	Vein	Single	NR	1 × 10^6^/kg	12
M. El-Ansary, 2010 [48]	Egypt	Uncontrolled	Chronic liver failure	12	49.67	75.0%	B-MSCs	Intrasplenic vs vein	Single	5	1 × 10^6^ cells	6
J. Chen, 2015 [49]	China	Uncontrolled	Decompensated liver cirrhosis	27	45.22	63.0%	UC-MSCs	Vein	Multiple	20	≥ 2 × 10^7^ cells	3
Z. Wang, 2013 [50]	China	Uncontrolled	Liver cirrhosis	10	53.7	90.0%	B-MSCs	Artery	Single	NR	1 × 10^6^/kg	3
L. Wang, 2013 [51]	China	Uncontrolled	Liver cirrhosis	10	49	10.0%	B-MSCs	Vein	Single	20	(0.5–1.0) × 10^6^/kg	12
Li. Wang, 2013 [52]	China	Uncontrolled	Decompensated liver cirrhosis	23	57.3	69.6%	UC-MSCs	Both vein and artery	Multiple	50	NR	12
H. Zhang, 2013 [53]	China	Uncontrolled	Decompensated liver cirrhosis	25	18–72 (range)	84.0%	B-MSCs	Artery	Single	3-5	(3–5) × 10^7^ cells	3
J. Li, 2012 [54]	China	Uncontrolled	Liver cirrhosis	87	46.9	65.5%	B-MSCs	Artery	Single	NR	1 × 10^6^ /kg	2
J. Wang, 2012 [55]	China	Uncontrolled	Decompensated liver cirrhosis	21	46	90.5%	UC-MSCs	Vein	Single	30	(0.6–1.5) × 10^9^ cells	3
J. Guo, 2012 [56]	China	Uncontrolled	Decompensated liver cirrhosis	15	32	66.7%	UC-MSCs	Both vein and artery	Multiple	20	(2–6) × 10^7^ cells	3
B. Zhou, 2011 [57]	China	Uncontrolled	Decompensated liver cirrhosis	60	57.97	58.3%	UC-MSCs	Artery	Single	40	2 × 10^7^ cells	3
H. Yu, 2011 [58]	China	Uncontrolled	Decompensated liver cirrhosis	10	6.78	100.0%	UC-MSCs	Artery	Single	NR	2 × 10^7^ cells	6
D. Niu, 2011 [59]	China	Uncontrolled	Autoimmune liver disease	7	53–68 (range)	28.6%	UC-MSCs	Vein	Multiple	NR	8 × 10^7^ cells	12
H. Chen, 2011 [60]	China	Uncontrolled	Chronic liver failure	20	45.2	70.0%	B-MSCs	Artery	Single	50	≥ 1 × 10^9^ cells	3
X. Jin, 2012 [61]	China	Uncontrolled	Decompensated liver cirrhosis	24	45.6	83.3%	UC-MSCs	Vein	Single	10	2 × 10^7^ cells	3

^a^ This nRCT was regarded as an uncontrolled trial because the control group data could not be extracted

*Artery* hepatic artery, *B-MSC* bone marrow-derived mesenchymal stem cell, *MSC* mesenchymal stem cell, *NR* not reported, *nRCT* nonrandomized controlled trial, *RCT* randomized controlled trial, *UC-MSC* umbilical cord-derived mesenchymal stem cell, *Vein* peripheral vein,

### Efficacy of MSC-based therapy and conventional supportive treatment

**Fig. 2:**
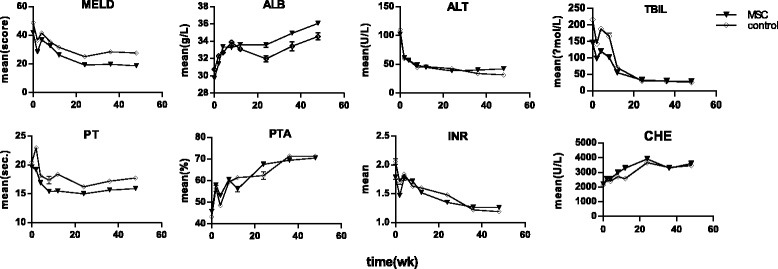
Changes in liver function from baseline to week 48. A total of 983 patients received mesenchymal stem cell (MSC)-based therapy, and 557 patients underwent conventional supportive treatment. After treatment, the model for end-stage liver disease (MELD) score, and the alanine aminotransferase (ALT), total bilirubin (TBiL), prothrombin time (PT), and international normalized ratio (INR) levels showed decreasing trends, while the albumin (ALB), prothrombin activity (PTA), and cholinesterase (CHE) levels showed increasing trends. Data are means ± SEM

A total of 983 patients received MSC-based therapy, and 557 patients underwent conventional supportive treatment. Changes in liver function from baseline to week 48 are shown in Fig. 2. After MSC-based therapy or conventional supportive therapy, the MELD score, and ALT, TBiL, PT, and INR values showed decreasing trends, while the ALB, PTA, and CHE levels showed increasing trends.

### Meta-analysis of the controlled trials

#### MELD

**Fig. 3:**
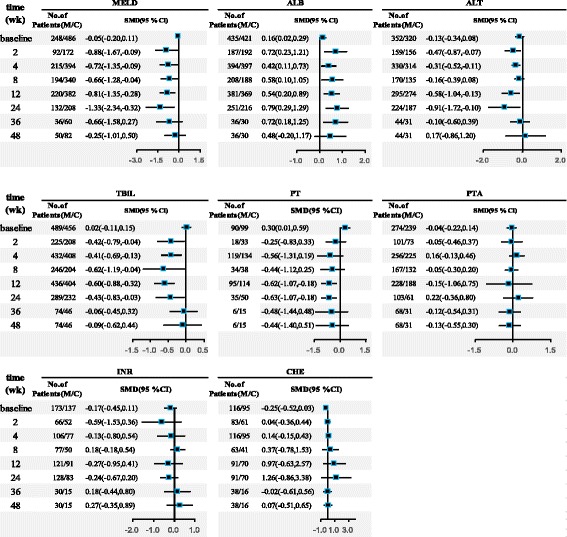
Forest plot of liver function. Compared with the control group (C), the model for end-stage liver disease (MELD) score in the MSC group (M) was nonsignificantly lower at baseline, significantly lower at weeks 2, 4, 8, 12, and 24, and nonsignificantly lower at weeks 36 and 48. The albumin (ALB) level in the MSC group was significantly higher at baseline and at weeks 2, 4, 8, 12, 24, and 36, and nonsignificantly higher at week 48. The alanine aminotransferase (ALT) level in the MSC group was nonsignificantly lower at baseline and at weeks 8, 36, and 48, and significantly lower at weeks 2, 4, 12, and 24. The total bilirubin (TBiL) level in the MSC group was non-significantly higher at baseline, significantly lower at weeks 2, 4, 8, 12, and 24, and non-significantly lower at weeks 36 and 48. The prothrombin time (PT) in the MSC group was significantly higher at baseline, non-significantly lower at weeks 2, 4, and 8, significantly lower at weeks 12 and 24, and non-significantly lower at weeks 36 and 48. The prothrombin activity (PTA), international normalized ratio (INR), and cholinesterase (CHE) values did not differ significantly between the MSC and control groups at any time point. CI confidence interval. SMD standardized mean difference

Eleven studies reported MELD scores during the follow-up period. At baseline, there was no significant difference between the MSC and control groups (SMD = −0.05, 95% CI −0.20 to 0.11; *I*^2^ = 0%, *P* = 0.73). After treatment, the magnitude of the decrease in the MELD score was greater in the MSC group compared with the control group and was statistically significant at week 2 (SMD = −0.88, 95% CI −1.67 to −0.09; *I*^2^ = 87%, *P* < 0.01), week 4 (SMD = −0.72, 95% CI −1.35 to −0.09; *I*^2^ = 91%, *P* < 0.01), week 8 (SMD = −0.66, 95% CI −1.28 to −0.04; *I*^2^ = 91%, *P* < 0.01), week 12 (SMD = −0.81, 95% CI −1.35 to −0.28; *I*^2^ = 88%, *P* < 0.01), and week 24 (SMD = −1.33, 95% CI −2.34 to −0.32; *I*^2^ = 93%, *P* < 0.01), but not at week 36 (SMD = −0.66, 95% CI −1.58 to 0.27; *I*^2^ = 68%, *P* = 0.08) or week 48 (SMD = −0.25, 95% CI −1.01 to 0.50; *I*^2^ = 73%, *P* = 0.03). These results are shown in Fig. 3.

Significant heterogeneity existed at most time points. Sensitivity analyses showed that one study [31] affected the heterogeneity the most (see Additional file 2). After excluding that study, the heterogeneity decreased at each time point. Publication bias was not assessed because of an insufficient number of studies.

#### ALB level

Eighteen studies reported ALB data during the follow-up period. After treatment, the magnitude of the increase in the ALB level was greater in the MSC group compared with the control group, and this difference was statistically significant at week 2 (SMD = 0.72, 95% CI 0.23 to 1.21; *I*^2^ = 79%, *P* < 0.01), week 4 (SMD = 0.42, 95% CI 0.11 to 0.73; *I*^2^ = 76%, *P* < 0.01), week 8 (SMD = 0.58, 95% CI 0.10 to 1.05; *I*^2^ = 80%, *P* < 0.01), week 12 (SMD = 0.54, 95% CI 0.20 to 0.89; *I*^2^ = 79%, *P* < 0.01), week 24 (SMD = 0.79, 95% CI 0.29 to 1.29; *I*^2^ = 84%, *P* < 0.01), and week 36 (SMD = 0.72, 95% CI 0.18 to 1.25; *I*^2^ = 0%, *P* = 0.81) after treatment, but not at week 48 (SMD = 0.48, 95% CI -0.20 to 1.17; *I*^2^ = 34%, *P* = 0.22). Additionally, the ALB level was significantly higher in the MSC group than in the control group at baseline (SMD = 0.16, 95% CI 0.02 to 0.29; *I*^2^ = 0%, *P* = 0.92). These results are shown in Fig. 3.

Significant heterogeneity existed at most time points. Sensitivity analyses showed that one study [31] affected the heterogeneity the most (see Additional file 3). After excluding that study, the heterogeneity of the remaining studies decreased at each time point. Publication bias was assessed at 4, 12, and 24 weeks, and asymmetry was observed at 12 and 24 weeks (see Additional file 4). Symmetrical contour-enhanced funnel plots combined with trim and fill analysis showed that, at week 12, four hypothetical studies were filled and plotted in the area of statistical significance (*i.e.*, the shaded area). At week 24, four hypothetical studies were filled: three plotted in the area of statistical significance and one in the area of statistical nonsignificance (*i.e.*, the nonshaded area). After filling, significance was lost at week 12 (SMD = 0.24, 95% CI −0.13 to 0.60; *P* = 0.21) and week 24 (SMD = 0.24, 95% CI −0.033 to 0.81; *P* = 0.41). The filled results are visualized in Additional file 5.

#### ALT level

Fourteen studies reported ALT data during the follow-up period. At baseline, there was no significant difference between the MSC and control groups (SMD = -0.13, 95% CI -0.34 to 0.08; *I*^2^ = 41%, *P* = 0.06). After treatment, the magnitude of the decrease in the ALT level was greater in the MSC group compared with the control group, and the difference was statistically significant at week 2 (SMD = −0.47, 95% CI −0.87, −0.07; *I*^2^ = 63%, *P* < 0.01), week 4 (SMD = −0.31, 95% CI −0.52 to −0.11; *I*^2^ = 39%, *P* = 0.08), week 12 (SMD = −0.58, 95% CI −1.04 to −0.13; *I*^2^ = 84%, *P* < 0.01), and week 24 (SMD = −0.91, 95% CI −1.72 to −0.10; *I*^2^ = 92%, *P* < 0.01), but not at week 8 (SMD = −0.16, 95% CI −0.39 to 0.08; *I*^2^ = 0%, *P* = 0.49) or week 36 (SMD = −0.10, 95% CI −0.60 to 0.39; *I*^2^ = 0%, *P* = 0.56). At week 48 after treatment, a higher ALT level was seen in the MSC group compared with the control group (SMD = 0.17, 95% CI −0.86 to 1.20; *I*^2^ = 70%, *P* = 0.07), but this was not statistically significant. These results are shown in Fig. 3.

Significant heterogeneity existed at some time points. Sensitivity analyses showed that two studies [31, 44] affected the heterogeneity the most (see Additional file 6). After excluding the results of Xu et al. [31] at week 2 and Zhuang et al. [44] at weeks 12 and 24, the heterogeneity of the remaining studies decreased at each time point. Publication bias was assessed at weeks 4 and 12; no significant publication bias was detected (see Additional file 7).

#### TBiL level

Nineteen studies reported TBiL data during the follow-up period. At baseline, no significant difference in the TBiL level was observed between the MSC and control groups (SMD = 0.02, 95% CI −0.11 to 0.15; *I*^2^ = 2%, *P* = 0.43). After treatment, the magnitude of the decrease in the TBiL level was greater in the MSC group than the control group, and the difference was significant at week 2 (SMD = −0.42, 95% CI −0.79 to −0.04; *I*^2^ = 69%, *P* < 0.01), week 4 (SMD = −0.41, 95% CI −0.69 to −0.13; *I*^2^ = 71%, *P* < 0.01), week 8 (SMD = −0.62, 95% CI −1.19 to −0.04; *I*^2^ = 87%, *P* < 0.01), week 12 (SMD = −0.60, 95% CI −0.88 to −0.32; *I*^2^ = 72%, *P* < 0.01), and week 24 (SMD = −0.43, 95% CI −0.83 to −0.03; *I*^2^ = 78%, *P* < 0.01), but not at week 36 (SMD = -0.06, 95% CI −0.45 to 0.32; *I*^2^ = 0%, *P* = 0.68) or week 48 (SMD = −0.09, 95% CI −0.62 to 0.44; *I*^2^ = 42%, *P* = 0.18). These results are shown in Fig. 3.

Significant heterogeneity existed at most time points. Sensitivity analyses were performed and showed that two studies [29, 31] affected the heterogeneity the most (see Additional file 8). After excluding the data from Xu et al. [31] at weeks 2, 4, and 24 and from Zhang et al. [29] at weeks 4 and 8, the heterogeneity of the remaining studies decreased at each time point. Publication bias was assessed at weeks 2, 4, 12, and 24, and asymmetry was detected at weeks 4 and 12 (see Additional file 9). Symmetrical contour-enhanced funnel plots combined with trim and fill analysis showed that, at week 4, four hypothetical studies were filled, with three plotted in the area of statistical significance and one in the area of statistical nonsignificance; at week 12, three hypothetical studies were filled, with two plotted in the area of statistical significance and one in the area of statistical nonsignificance. After filling, significance was lost at week 4 (SMD = −0.11, 95% CI −0.44 to 0.21; *P* = 0.50) but not at week 12 (SMD = −0.38, 95% CI −0.70 to −0.06; *P* = 0.02). The filled results are visualized in Additional file 10.

#### PT

Five studies reported PT data during the follow-up period. The PT in the MSC group was significantly higher at baseline (SMD = 0.30, 95% CI 0.01 to 0.59; *I*^2^ = 0%, *P* = 0.92), nonsignificantly lower at week 2 (SMD = −0.25, 95% CI −0.83 to 0.33; *I*^2^ = 0%, *P* = 0.58), week 4 (SMD = −0.56, 95% CI −1.31 to 0.19; *I*^2^ = 86%, *P* < 0.01), and week 8 (SMD = −0.44, 95% CI −1.12 to 0.25; *I*^2^ = 50%, *P* = 0.16), significantly lower at week 12 (SMD = −0.62, 95% CI −1.07 to −0.18; *I*^2^ = 51%, *P* = 0.11) and week 24 (SMD = −0.63, 95% CI −1.07 to −0.18; *I*^2^ = 0%, *P* = 0.60), and nonsignificantly lower at week 36 (SMD = −0.48, 95% CI −1.44 to 0.48) and week 48 (SMD = −0.44, 95% CI −1.40 to 0.51). The results are shown in Fig. 3. Publication bias was not assessed because of an insufficient number of studies.

#### PTA, INR, and CHE levels

Eleven studies reported PTA data during the follow-up period. No significant difference was observed in PTA between the MSC and control groups at baseline or any time point after treatment. Moreover, the INR (six studies) and CHE (six studies) levels did not differ significantly between the two groups at any time point. These results are shown in Fig. 3.

### Subgroup meta-analysis

#### Study design (nRCT or RCT)

##### MELD score and ALB, ALT, and TBiL levels

**Fig. 4:**
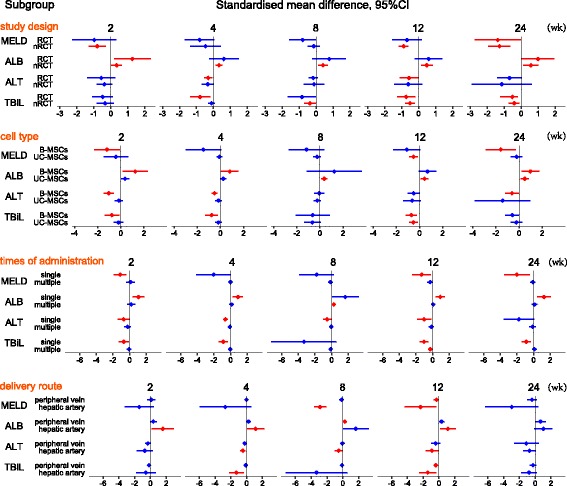
Meta-analysis of patient subgroups**.** Red indicates significant improvement in the mesenchymal stem cell (MSC) group compared with the control group; blue indicates no significant improvement. Subgroup analyses at other time points and of other parameters could not be conducted because of an insufficient number of studies. ALB albumin, ALT alanine aminotransferase, B-MSC bone marrow-derived mesenchymal stem cell, CI confidence interval, MELD model for end-stage liver disease, nRCT nonrandomized controlled trial, RCT randomized controlled trial, TBiL total bilirubin, UC-MSC umbilical cord-derived mesenchymal stem cell

Compared with the control group, the nRCT-MSC group showed a significant reduction in the MELD score at weeks 2, 12, and 24 after treatment, while the RCT-MSC group showed a significant reduction at week 24. The nRCT MSC group showed a significant increase in ALB levels at weeks 2, 4, 8, 12, and 24 after treatment, while the RCT-MSC group showed a significant increase at weeks 2 and 24. The nRCT-MSC group showed no significant reduction in ALT levels at any time point, while the RCT-MSC group showed a significant reduction in ALT levels at weeks 4 and 12 after treatment. The nRCT-MSC group showed a significant reduction in TBiL levels at weeks 8, 12, and 24 after treatment, while the RCT-MSC group showed a significant reduction at weeks 4, 12 and 24. These results are shown in Fig. 4.

#### Cell type (B-MSCs or UC-MSCs)

##### MELD score and ALB, ALT, and TBiL levels

Compared with the control group, the B-MSC group showed a significant reduction in the MELD score at weeks 2 and 24 after treatment, while the UC-MSC group showed a significant reduction at week 12 only. The B-MSC group showed a significant increase in ALB levels at weeks 2, 4, and 24 after treatment, while the UC-MSC group showed a significant increase at weeks 8, 12, and 24. The B-MSC group showed a significant reduction in ALT levels at weeks 2, 4, and 24 after treatment, while the UC-MSC group showed no significant reduction at any time point. The B-MSC group showed a significant reduction in TBiL levels at weeks 2, 4, and 12 after treatment, while the UC-MSC group showed a significant reduction at week 12 only. These results are shown in Fig. 4.

#### Frequency of administration (single or multiple injection)

##### MELD score and ALB, ALT, and TBiL levels

Compared with the control group, the single MSC injection group showed a significant reduction in MELD score at weeks 2, 12, and 24 after treatment, while the multiple MSC injection group showed no significant reduction at any time point. The single MSC injection group showed a significant increase in ALB levels at weeks 2, 4, 12, and 24 after treatment, while the multiple MSC injection group showed a significant increase at week 8 only. The single MSC injection group showed a significant reduction in ALT levels at weeks 2, 4, 8, and 12 after treatment, while the multiple MSC injection group showed no significant reduction at any time point. The single MSC injection group showed a significant reduction in TBiL levels at weeks 2, 4, 12, and 24 after treatment, while the multiple MSC injection group showed a significant reduction at week 12 only. These results are shown in Fig. 4.

#### Delivery route (peripheral vein or hepatic artery)

##### MELD score and ALB, ALT, and TBiL levels

Compared with the control group, the hepatic artery administration group showed a significant reduction in the MELD score at weeks 8 and 12 after treatment, while the peripheral vein administration group showed a significant reduction at week 12 only. The hepatic artery administration group showed a significant increase in ALB levels at weeks 2, 4, and 12 after treatment, while the peripheral vein administration group showed a significant increase at week 8 only. The hepatic artery administration group showed a significant reduction in ALT levels at weeks 4, 8, and 12 after treatment, while the peripheral vein administration group showed no significant reduction at any time point. The hepatic artery administration group showed a significant reduction in TBiL levels at weeks 4 and 12 after treatment, while the peripheral vein administration group showed a significant reduction at week 12 only. These results are shown in Fig. 4.

### Procedural adverse events and complications

The procedural adverse events reported by the 23 controlled trials included fever [25, 27, 29, 31, 33, 35–37, 41, 42, 44, 45], transient shivering [27], nausea [41], ecchymosis [34, 41], local pain [34, 36], rash [35], and diarrhea [31–35]. All of these adverse events resolved spontaneously. Six studies reported complications, *i.e.*, gastrointestinal bleeding [25, 28, 35], primary peritonitis [25, 28], hepatic encephalopathy [25, 28, 34, 35, 43], acute cholecystitis [25], infection [34, 35, 43, 44], hepatorenal syndrome [35, 43], and hepatocellular carcinoma [25, 28, 34] in the MSC and control groups. The difference in the incidence of adverse events or complications between the two groups was not significant in four studies [25, 28, 34, 44], while the incidences of infection [35] and hepatic encephalopathy [43] were markedly lower in the MSC group than the control group.

## Discussion

This study was a meta-analysis of 23 controlled trials, comprising both RCTs and nRCTs. MSC therapy improved the MELD score, ALB, ALT, and TBiL levels, and PT of patients with liver disease. However, no improvement in PTA, INR, or CHE levels was evident.

To verify our results, a subgroup meta-analysis of RCTs and nRCTs was conducted. The MELD score and ALB level improved significantly at more time points in the nRCT subgroup than the RCT subgroup. However, a significant improvement in the ALT level was detected only in the RCT subgroup. The TBiL level improved significantly at more time points in the nRCT subgroup but at earlier time points in the RCT subgroup. Nevertheless, at week 24, the MELD score, ALB, ALT, and TBiL levels were similar between the nRCT and RCT subgroups.

To identify factors related to the efficacy of MSC therapy, we conducted subgroup meta-analyses according to cell type, MSC administration frequency, and MSC delivery route.

The efficacy of stem cell therapy in patients with liver disease has long been discussed. Among the candidate cell types, MSCs are suggested to be superior to hematopoietic stem cells for treating cirrhosis [63, 64]. MSCs are readily available from a variety of tissues, *e.g.*, bone marrow, umbilical cord blood, adipose tissue, and placenta. The studies in our meta-analysis used B-MSCs or UC-MSCs. Although discontinuous, significant improvements in all four liver function parameters occurred at more time points in the B-MSC subgroup than the UC-MSC subgroup. This may be due to differences in homing ability among MSCs from different tissues [65].

Our data confirm a previous report that the beneficial effect of MSC administration is not prolonged [66]. For those improvements that disappeared at week 36 (MELD score, ALT and TBiL levels, and PT) or week 48 (ALB level), the fact that few of the included studies involved such a long follow-up period should be taken into account. A previous meta-analysis [66] suggested that re-administration prolongs the efficacy of MSC therapy; however, our subgroup analysis of multiple MSC injection revealed no evidence of such a benefit. In contrast, a single administration exerted a greater beneficial effect, particularly on the MELD score and ALT level. The interval between the first and second administrations was not analyzed in our study because of an inadequate number of trials.

Although intravenous injection is the most common mode of MSC administration [67], intra-arterial injection exhibited greater efficacy in this meta-analysis. Significant improvements in liver function occurred earlier (MELD score and ALB and TBiL levels), or only (ALT level), in the hepatic artery administration group compared with the peripheral vein administration group. Although discontinuous among time points, the improvements were significant at more time points in the hepatic artery administration group. Walczak et al. [68] reported that intra-arterial administration significantly enhances homing of MSCs to the site of injury compared with distant intravenous administration. Intravenously administered MSCs may be phagocytosed by reticuloendothelial cells in capillary-rich tissues. Following intravenous injection, MSCs initially travel to the lungs, followed by the liver; subsequently, the signal decreases over time, as seen in animal studies [69, 70]. Thus, only a portion of the MSCs injected intravenously reach the injured tissue. Moreover, the number of MSCs injected likely influences the therapeutic efficacy. For example, the optimal number of MSCs may differ between intra-arterial and intravenous administrations.

Publication bias states that studies reporting unfavorable or uninteresting results are less likely to be published [71]. Asymmetry was observed in funnel plots at several time points; further analysis suggested the possibility of publication bias. However, publication bias is not the only possible cause of asymmetry in funnel plots [72]. Most of the filled studies were plotted in the area of statistical significance; thus, the asymmetry was largely due to other factors. Any factor associated with both study effect and study size could lead to an asymmetric funnel plot [22]. The significant heterogeneity detected in this meta-analysis is one such factor.

Considerable heterogeneity existed at most time points, for which a subgroup meta-analysis according to cell type, delivery route, administration frequency, and type of trial did not provide a clear explanation. The sensitivity analyses suggested that the heterogeneity could be decreased by excluding the studies of Xu et al. [31], Zhuang et al. [44], or Zhang et al. [29]. In particular for time-to-event outcomes, lack of adjustment for censoring leads to an imprecise estimate of the overall treatment effect and interstudy heterogeneity [73]. Moreover, marked variation in the study characteristics and the degree of progression of liver disease might also be sources of heterogeneity among the included studies. An ongoing challenge in the discipline is variation in the techniques by which MSCs are isolated, cultured, and manipulated *in vitro*, timing and dose of MSC administration, and the follow-up duration. These discrepancies are likely to account for some of the heterogeneity among studies, suggesting a need for greater consensus regarding standards.

Two other limitations must also be considered. First, the improvement in the ALB level is probably inadequate because the baseline value was significantly higher in the MSC group than the control group. Although the significant improvement in ALB level was maintained until week 36—the longest of all liver function parameters evaluated—this must be verified since the trim-and-fill sensitivity analysis altered this result at two time points. Second, the outcome parameters and measurement time points used by the included studies were not consistent. Therefore, the number of pooled studies that could be used to assess an outcome at a particular time point, especially longer time points, was limited. These limitations are likely to cause unreliability of the study; thus, the conclusions drawn in this manuscript should be further verified or refined by integrating other available data if more relevant literature is published in the near future. Nevertheless, our meta-analysis is of interest because we determined the optimal administration frequency, delivery route, and source of MSCs.

MSCs coordinate dynamic and integrated hepatic reparative effects as follows: 1) differentiation into hepatocytes; 2) suppression of immune reactions; 3) suppression of fibrosis; and 4) inhibition of hepatocellular apoptosis and stimulation of liver regeneration [74, 75]. The beneficial effect of MSCs is mediated principally by paracrine mechanisms involving various bioactive molecules, including growth factors and cytokines [76]. These molecules reduce liver inflammation and fibrosis and replenish functional hepatocytes, preventing progressive distortion of the hepatic architecture. Although evidence of multiple functional roles for MSC administration has been found, the effectiveness of MSC therapy is affected by diverse factors. Due to the limited number of included studies and inconsistent outcome indicators, we performed four stratified analyses of relevant variables. However, other variables, *e.g.*, the timing and the number of cells injected, are also important considerations for MSC therapy. A sufficient number of further high-quality clinical trials are required to investigate these unknown factors and establish standards.

## Conclusions

The results of this meta-analysis show that MSC administration improves the MELD score, ALT and TBiL levels, and PT of patients with liver disease. A single MSC administration, administration into the hepatic artery, and use of B-MSCs were optimal in terms of improving hepatic function. The long-term effect of MSC administration and the discontinuous results of the subgroup meta-analysis should be the subject of further investigation. Robust evidence is lacking because of the significant heterogeneity among studies. Therefore, further high-quality studies using currently available standardized measures are needed. Establishment of guidelines and protocols for MSC-based therapy in future clinical trials will facilitate the use of MSCs as a safe and effective therapy for patients with liver disease.

## Additional files

Additional file 1: Search strategy. (PDF 52 kb)

Additional file 2: Results of sensitivity analyses: MELD. (PDF 47 kb)

Additional file 3: Results of sensitivity analyses: ALB levels. (PDF 52 kb)

Additional file 4: Visualized results of publication bias of ALB. (PDF 120 kb)

Additional file 5: Results of symmetrical contour-enhanced funnel plots combined with trim and fill analysis of ALB. (PDF 165 kb)

Additional file 6: Results of sensitivity analyses: ALT levels. (PDF 41 kb)

Additional file 7: Visualized results of publication bias of ALT. (PDF 106 kb)

Additional file 8: Results of sensitivity analyses: TBiL levels. (PDF 54 kb)

Additional file 9: Visualized results of publication bias of TBiL. (PDF 133 kb)

Additional file 10: Results of symmetrical contour-enhanced funnel plots combined with trim and fill analysis of TBiL. (PDF 167 kb)
