# Supplementary material for: A pooled analysis of mesenchymal stem cell-based therapy for liver disease
Source: Stem Cell Res Ther. 2018 Mar 21;9:72. doi: 10.1186/s13287-018-0816-2 (PMC5863358; doi:10.1186/s13287-018-0816-2)
Supplement: Supplementary file 1 — Search strategy. (PDF 52 kb) [file 13287_2018_816_MOESM1_ESM.pdf]

Web appendix

Search strategy:

Medline (PubMed)
#1 liver
#2 liver [MeSH Terms]
#3 liver diseases [MeSH Terms]
#4 #1 or #2 or #3
#5 “mesenchymal stem cell*”
#6 “multipotent stromal cell*”
#7"Mesenchymal Stromal Cells"[Mesh]
#8 "Mesenchymal Stem Cell Transplantation"[Mesh]
#9 #5 or #6 or #7 or #8
#10 #4 and #9 filters: clinical trial

Cochrane Library
#1 MeSH descriptor: [Mesenchymal Stromal Cells] explode all trees
#2 “mesenchymal stem cell*” or “multipotent stromal cell*”
#3 (#1 or #2)
#4 MeSH descriptor: [Liver Diseases] explode all trees
#5 hepatitis or "hepatic fibrosis" or "liver fibrosis" or "liver cirrhosis" or "Liver Neoplasm*" or "Liver Failure" or "Fatty Liver" or "Liver Abscess" or "Liver Injury"
#6 liver
#7 MeSH descriptor: [Liver] explode all trees
#8 （#4 or #5 or #6 or #7）
#9（#3 and #8）

EMBASE
#1 “mesenchymal stem cell”/exp OR “mesenchymal stem cell”
#2 “liver” /exp OR “liver”
#3 #1 AND #2

SinoMed CBM
(((“mesenchymal stem cell*” [unweighted: extension]) OR “multipotent stromal cell*” [unweighted: extension]) OR “totipotential stem cell" [unweighted: extension]) AND “liver diseases” [unweighted: extension]

linicalTrials.gov
“Mesenchymal stem cells” and “liver” |studies with Results
