# Supplementary material for: A pooled analysis of mesenchymal stem cell-based therapy for liver disease
Source: Stem Cell Res Ther. 2018 Mar 21;9:72. doi: 10.1186/s13287-018-0816-2 (PMC5863358; doi:10.1186/s13287-018-0816-2)
Supplement: Supplementary file 2 — Results of sensitivity analyses: MELD. (PDF 47 kb) [file 13287_2018_816_MOESM2_ESM.pdf]

Results of sensitivity analyses with omission of one study at a time:

***** Sensitivity analyses were not performed at weeks 36 and 48 because there were just two studies.

--MELD

Week 2

##Influential analysis (Random effects model)

##

##	SMD 95%-CI	p-value	tau^2	I^2
## Omitting Amer M E,2011	-0.9096 [-1.9709; 0.1517]	0.0930	1.0503	90.40%
## Omitting L. Peng, 2011	-0.9746 [-1.9174; -0.0317]	0.0428	0.8323	90.20%
**## Omitting L. Xu, 2014**	**-0.5199 [-1.0478; 0.0080]**	**0.0536**	**0.1852**	**65.10%**
## Omitting Z. Zhang, 2012	-1.1466 [-1.9036; -0.3895]	0.0030	0.4721	80.00%
## Omitting B. Liu, 2013	-0.8586 [-1.8763; 0.1592]	0.0983	0.9620	90.20%
##				
## Pooled estimate	-0.8807 [-1.6706; -0.0907]	0.0289	0.6992	87.20%

Week 4

Influential analysis (Random effects model)

##

##	SMD 95%-CI	p-value	tau^2	I^2
## Omitting Amer M E,2011	-0.7162 [-1.4277; -0.0047]	0.0485	0.8024	92.10%
## Omitting Lin B-L,2017	-0.8723 [-1.6381; -0.1065]	0.0256	0.9390	91.80%
## Omitting L. Peng, 2011	-0.6818 [-1.3627; -0.0009]	0.0497	0.7434	92.20%
**## Omitting L. Xu, 2014**	**-0.2015 [-0.4685; 0.0655]**	**0.1392**	**0.0577**	**48.20%**
## Omitting Z. Zhang, 2012	-0.8296 [-1.5654; -0.0937]	0.0271	0.8637	92.50%
## Omitting H. He, 2015	-0.8425 [-1.6335; -0.0514]	0.0369	1.0102	92.40%
## Omitting Y. Li, 2015	-0.8377 [-1.5896; -0.0858]	0.0290	0.9042	92.40%
## Omitting Q. Wang, 2013	-0.7888 [-1.4759; -0.1016]	0.0245	0.7641	92.50%
## Pooled estimate	-0.7188 [-1.3523; -0.0852]	0.0262	0.7218	91.30%

Week 8

Influential analysis (Random effects model)

##

##	SMD 95%-CI	p-value	tau^2	I^2
## Omitting Amer M E,2011	-0.6417 [-1.3635; 0.0802]	0.0815	0.6131	92.10%
## Omitting Lin B-L,2017	-0.8231 [-1.5749; -0.0712]	0.0319	0.6612	91.30%
**## Omitting L. Xu, 2014**	**-0.2559 [-0.5586; 0.0467]**	**0.0974**	**0.0687**	**58.60%**
## Omitting Z. Zhang, 2012	-0.7999 [-1.5342; -0.0657]	0.0327	0.6339	92.10%
## Omitting H. He,2015	-0.7881 [-1.5883; 0.0121]	0.0536	0.7577	92.10%
## Omitting Y. Li,2015	-0.6879 [-1.4500; 0.0742]	0.0769	0.6851	92.30%
##				
## Pooled estimate	-0.6587 [-1.2797; -0.0376]	0.0376	0.5351	90.60%

Week 12

Influential analysis (Random effects model)

##	SMD 95%-CI	p-value	tau^2	I^2
## Omitting Lin B-L,2017	-0.9292 [-1.5316; -0.3269]	0.0025	0.6359	87.60%
## Omitting Mehdi M, 2013	-0.9271 [-1.5058; -0.3485]	0.0017	0.5892	88.70%
## Omitting L. Peng, 2011	-0.7632 [-1.3312; -0.1951]	0.0085	0.5752	88.90%
**## Omitting L. Xu, 2014**	**-0.4820 [-0.8126; -0.1514]**	**0.0043**	**0.1374**	**65.40%**
## Omitting Z. Zhang, 2012	-0.9205 [-1.5173; -0.3238]	0.0025	0.6264	88.60%
## Omitting H. He,2015	-0.8382 [-1.4877; -0.1887]	0.0114	0.7574	89.10%
## Omitting Y. Li,2015	-0.8884 [-1.5128; -0.2640]	0.0053	0.6940	89.20%
## Omitting B. Liu, 2013	-0.7841 [-1.3656; -0.2026]	0.0082	0.5983	89.00%
## Omitting El-Ansary M,2012	-0.7896 [-1.3664; -0.2129]	0.0073	0.5928	89.10%
##				
## Pooled estimate	-0.8131 [-1.3484; -0.2778]	0.0029	0.5625	87.80%

Week 24

Influential analysis (Random effects model)

##	SMD 95%-CI	p-value	tau^2	I^2
## Omitting Amer M E,2011	-1.4342 [-2.7186; -0.1497]	0.0286	1.9876	94.60%
## Omitting Lin B-L,2017	-1.6277 [-2.8746; -0.3807]	0.0105	1.8552	93.10%
## Omitting L. Peng, 2011	-1.3549 [-2.5151; -0.1947]	0.0221	1.6213	94.60%
**## Omitting L. Xu, 2014**	**-0.6481 [-1.1660; -0.1303]**	**0.0142**	**0.2394**	**72.50%**
## Omitting Z. Zhang, 2012	-1.5855 [-2.8822; -0.2887]	0.0166	2.0255	94.40%
## Omitting El-Ansary M,2012	-1.3423 [-2.5044; -0.1803]	0.0236	1.6237	94.50%
##				
## Pooled estimate	-1.3308 [-2.3428; -0.3188]	0.0100	1.4543	93.40%
