# Supplementary material for: A pooled analysis of mesenchymal stem cell-based therapy for liver disease
Source: Stem Cell Res Ther. 2018 Mar 21;9:72. doi: 10.1186/s13287-018-0816-2 (PMC5863358; doi:10.1186/s13287-018-0816-2)
Supplement: Supplementary file 3 — Results of sensitivity analyses: ALB levels. (PDF 52 kb) [file 13287_2018_816_MOESM3_ESM.pdf]

Results of sensitivity analyses with omission of one study at a time:

***** Sensitivity analyses were not performed at weeks 36 and 48 because there were just two studies.

--ALB level

Week 2

Influential analysis (Random effects model)

##

##	SMD 95%-CI	p-value	tau^2	I^2
## Omitting L. Peng, 2011	0.7263 [0.1880; 1.2645]	0.0082	0.4823	81.90%
## Omitting Salama H,2014	0.6769 [0.1326; 1.2211]	0.0148	0.4819	80.80%
**## Omitting L. Xu, 2014**	**0.4612 [0.1623; 0.7601]**	**0.0025**	**0.0730**	**40.50%**
## Omitting Z. Zhang, 2012	0.8008 [0.2515; 1.3501]	0.0043	0.4912	80.80%
## Omitting B. Liu, 2013	0.7590 [0.2022; 1.3157]	0.0075	0.5115	81.80%
## Omitting H. Luo,2015	0.7594 [0.1539; 1.3649]	0.0140	0.6167	81.90%
## Omitting L. Tong,2015	0.8454 [0.3413; 1.3494]	0.0010	0.3932	77.10%
## Omitting J. Yu,2012	0.7764 [0.2232; 1.3297]	0.0059	0.5033	81.60%
## Omitting Y. Zhang,2012	0.6898 [0.1473; 1.2322]	0.0127	0.4830	81.40%
##				
## Pooled estimate	0.7187 [0.2254; 1.2120]	0.0043	0.4378	79.30%

Week 4

Influential analysis (Random effects model)

##

##	SMD 95%-CI	p-value	tau^2	I^2
## Omitting H. Luo,2015	0.4188 [0.0805; 0.7571]	0.0153	0.3046	77.20%
## Omitting H. He,2015	0.4182 [0.0794; 0.7569]	0.0155	0.3055	77.10%
## Omitting J. Yu,2012	0.4427 [0.1187; 0.7668]	0.0074	0.2776	77.10%
## Omitting Lin B-L,2017	0.4619 [0.1345; 0.7893]	0.0057	0.2788	75.50%
## Omitting L. Zhuang,2014	0.4214 [0.0888; 0.7540]	0.0130	0.2931	77.30%
## Omitting L. Tong,2015	0.4590 [0.1377; 0.7803]	0.0051	0.2697	76.40%
## Omitting M. Zhu,2013	0.4069 [0.0815; 0.7324]	0.0143	0.2793	77.00%
## Omitting L. Peng, 2011	0.3903 [0.0757; 0.7049]	0.0150	0.2625	76.60%
## Omitting Salama H,2014	0.3534 [0.0520; 0.6548]	0.0216	0.2277	73.50%
## Omitting S. Ouyang,2013	0.4464 [0.1149; 0.7778]	0.0083	0.2900	77.00%
**## Omitting L. Xu, 2014**	**0.2891 [0.0568; 0.5213]**	**0.0147**	**0.1033**	**56.20%**
## Omitting Y. Li,2015	0.4528 [0.1253; 0.7802]	0.0067	0.2814	76.70%
## Omitting Y. Zhang,2012	0.3956 [0.0760; 0.7152]	0.0153	0.2691	76.80%
## Omitting Z. Zhang, 2012	0.4746 [0.1619; 0.7873]	0.0029	0.2506	75.10%
## Omitting Q. Wang,2013	0.4707 [0.1634; 0.7780]	0.0027	0.2472	75.60%
##				
## Pooled estimate	0.4191 [0.1128; 0.7254]	0.0073	0.2605	75.60%

Week 8

Influential analysis (Random effects model)
##

##	SMD 95%-CI	p-value	tau^2	I^2
## Omitting H. He,2015	0.6162 [0.0117; 1.2207]	0.0457	0.4643	83.10%
## Omitting Lin B-L,2017	0.6852 [0.1283; 1.2421]	0.0159	0.3804	80.80%
## Omitting M. Zhu,2013	0.5822 [0.0291; 1.1352]	0.0391	0.3831	82.90%
**## Omitting L. Xu, 2014**	**0.3025 [0.0637; 0.5413]**	**0.0130**	**0.0162**	**18.10%**
## Omitting Y. Li,2015	0.6622 [0.0996; 1.2248]	0.0211	0.3942	82.40%
## Omitting Y. Zhang,2012	0.5489 [0.0178; 1.0799]	0.0428	0.3535	82.50%
## Omitting Z. Zhang, 2012	0.6852 [0.1531; 1.2174]	0.0116	0.3480	81.50%
##				
## Pooled estimate	0.5769 [0.1005; 1.0533]	0.0176	0.3189	79.80%

Week 12

Influential analysis (Random effects model)
##

##	SMD 95%-CI	p-value	tau^2	I^2
## Omitting B. Liu, 2013	0.5123 [0.1573; 0.8673]	0.0047	0.3497	80.20%
## Omitting H. Luo,2015	0.5897 [0.2136; 0.9657]	0.0021	0.3945	80.20%
## Omitting H. He,2015	0.5746 [0.1914; 0.9577]	0.0033	0.4136	80.90%
## Omitting Lin B-L,2017	0.6124 [0.2601; 0.9647]	0.0007	0.3335	77.90%
## Omitting L. Zhuang,2014	0.5108 [0.1522; 0.8694]	0.0052	0.3510	79.30%
## Omitting L. Tong,2015	0.5527 [0.1848; 0.9207]	0.0032	0.3779	80.90%
## Omitting Mehdi M, 2013	0.6210 [0.2787; 0.9633]	0.0004	0.3178	78.60%
## Omitting L. Peng, 2011	0.5043 [0.1539; 0.8546]	0.0048	0.3421	80.00%
## Omitting Salama H,2014	0.4919 [0.1435; 0.8403]	0.0057	0.3291	78.90%
## Omitting S. Ouyang,2013	0.5868 [0.2150; 0.9586]	0.0020	0.3845	80.50%
**## Omitting L. Xu, 2014**	**0.4308 [0.1207; 0.7409]**	**0.0065**	**0.2424**	**73.60%**
## Omitting Y. Li,2015	0.6050 [0.2458; 0.9643]	0.0010	0.3526	79.30%
## Omitting Y. Zhang,2012	0.5092 [0.1544; 0.8640]	0.0049	0.3477	80.00%
## Omitting Z. Zhang, 2012	0.5722 [0.2043; 0.9400]	0.0023	0.3775	80.90%
## Omitting El-Ansary M,2012	0.4944 [0.1461; 0.8426]	0.0054	0.3337	79.50%
##				
## Pooled estimate	0.5443 [0.2003; 0.8882]	0.0019	0.3479	79.50%

Week 24

Influential analysis (Random effects model)
##

##	SMD 95%-CI	p-value	tau^2	I^2
## Omitting Lin B-L,2017	0.9122 [0.3852; 1.4391]	0.0007	0.5177	82.30%
## Omitting L. Zhuang,2014	0.8145 [0.2405; 1.3885]	0.0054	0.6392	85.50%
## Omitting L. Tong,2015	0.8584 [0.3003; 1.4166]	0.0026	0.6018	85.40%
## Omitting L. Peng, 2011	0.7790 [0.2442; 1.3138]	0.0043	0.5576	85.40%
## Omitting Salama H,2014	0.6839 [0.1865; 1.1812]	0.0070	0.4578	82.20%

## Omitting S. Ouyang,2013	0.8918 [0.3355; 1.4481]	0.0017	0.5918	84.20%
## Omitting Suk KT,2017	0.8608 [0.2967; 1.4248]	0.0028	0.6150	85.40%
**## Omitting L. Xu, 2014**	**0.5496 [0.1674; 0.9318]**	**0.0048**	**0.2327**	**70.80%**
## Omitting Z. Zhang, 2012	0.8485 [0.2886; 1.4085]	0.0030	0.6065	85.50%
## Omitting El-Ansary M,2012	0.7360 [0.2106; 1.2614]	0.0060	0.5307	84.60%
##				
## Pooled estimate	0.7923 [0.2914; 1.2932]	0.0019	0.5287	83.80%
