# Supplementary material for: A pooled analysis of mesenchymal stem cell-based therapy for liver disease
Source: Stem Cell Res Ther. 2018 Mar 21;9:72. doi: 10.1186/s13287-018-0816-2 (PMC5863358; doi:10.1186/s13287-018-0816-2)
Supplement: Supplementary file 4 — Visualized results of publication bias of ALB. (PDF 120 kb) [file 13287_2018_816_MOESM4_ESM.pdf]

Visualized results of publication bias of ALB

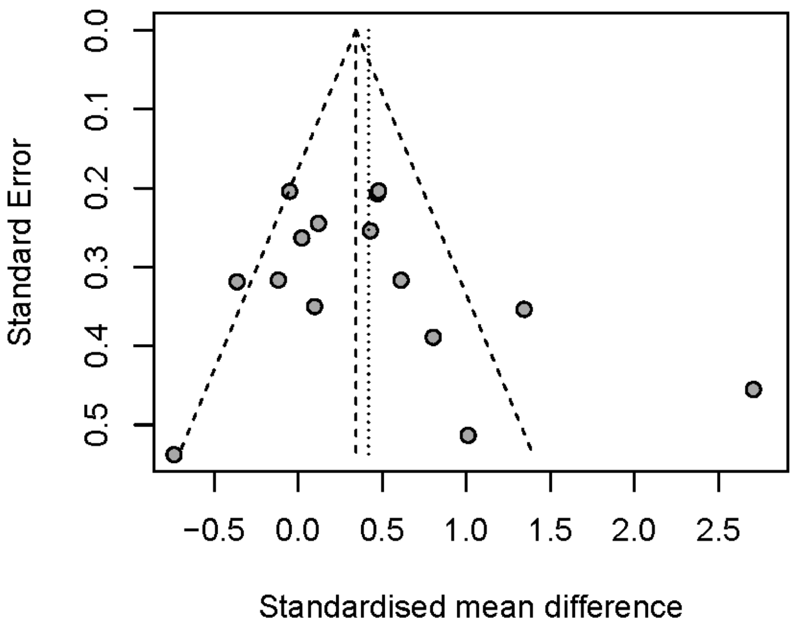

Figure S1. Bias assessment plot for ALB level at week 4

**p=0.28*

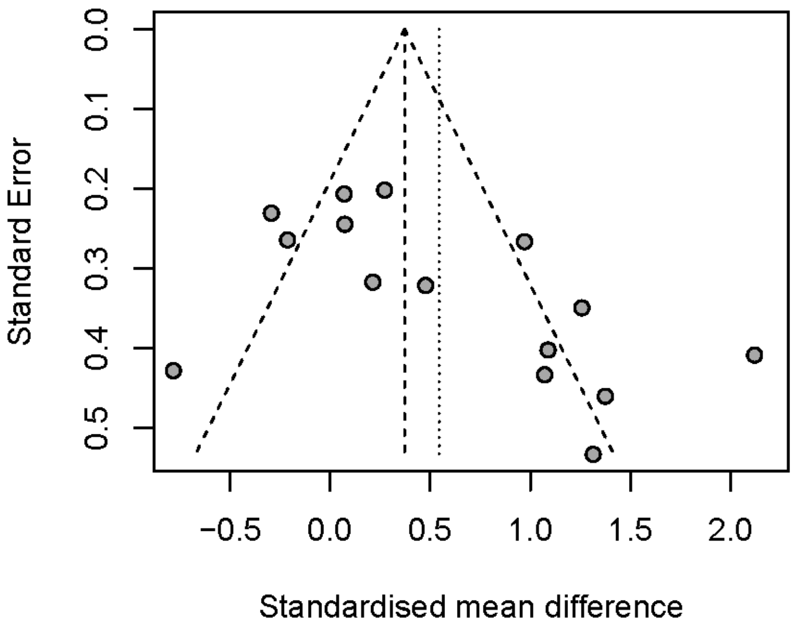

Figure S2. Bias assessment plot for ALB level at week 12

**p=0.03*

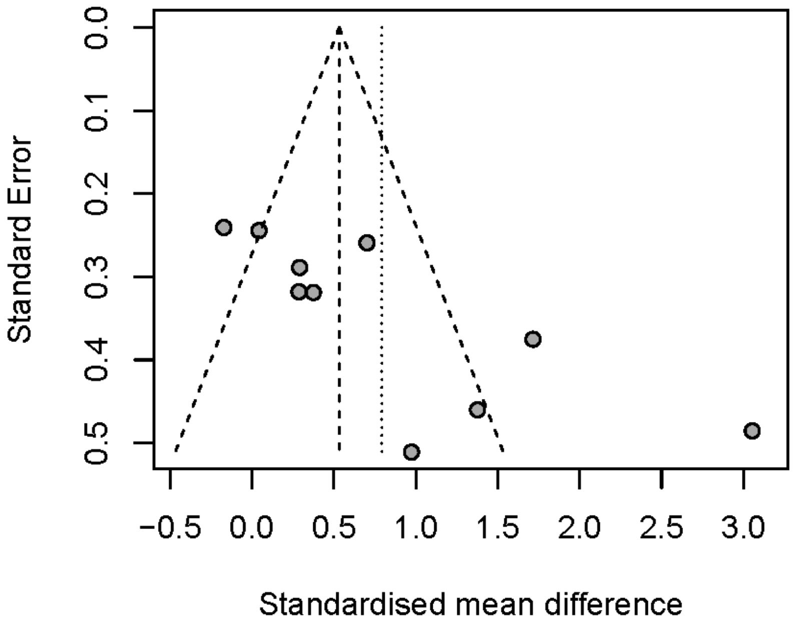

Figure S3. Bias assessment plot for ALB level at week 24

** p<0.01*
