# Supplementary material for: A pooled analysis of mesenchymal stem cell-based therapy for liver disease
Source: Stem Cell Res Ther. 2018 Mar 21;9:72. doi: 10.1186/s13287-018-0816-2 (PMC5863358; doi:10.1186/s13287-018-0816-2)
Supplement: Supplementary file 5 — Results of symmetrical contour-enhanced funnel plots combined with trim and fill analysis of ALB. (PDF 165 kb) [file 13287_2018_816_MOESM5_ESM.pdf]

Results of symmetrical contour-enhanced funnel plots combined with trim and fill analysis of ALB:

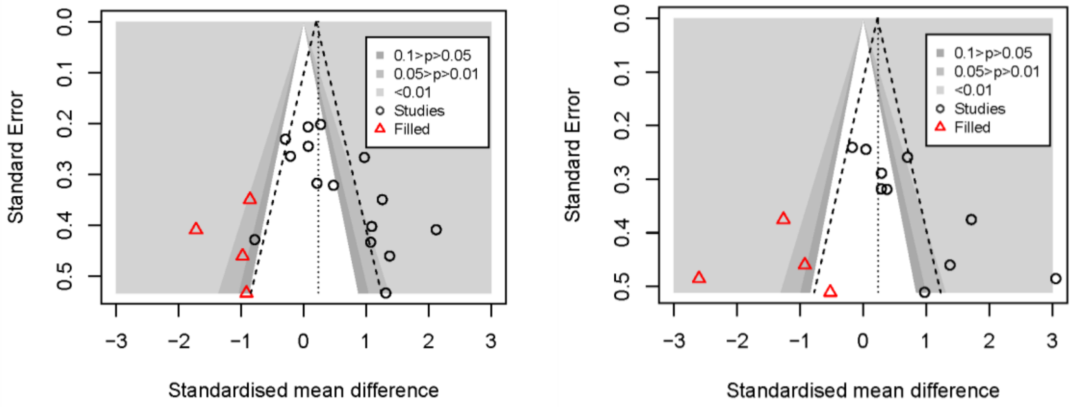

Figure S10 Symmetrical contour-enhanced funnel plots for ALB at weeks 12 and 24

At week 12, four hypothetical studies were filled, and all plotted in the area of statistical significant (*i.e.*, the shaded area), indicating that the asymmetry at week 12 was not caused by publication bias; at the week 24, four hypothetical studies were filled: three plotted in the area of statistical significant and one in the area of statistical nonsignificant, indicating that the asymmetry at week 24 was partly caused by publication bias.
