# Supplementary material for: A pooled analysis of mesenchymal stem cell-based therapy for liver disease
Source: Stem Cell Res Ther. 2018 Mar 21;9:72. doi: 10.1186/s13287-018-0816-2 (PMC5863358; doi:10.1186/s13287-018-0816-2)
Supplement: Supplementary file 6 — Results of sensitivity analyses: ALT levels. (PDF 41 kb) [file 13287_2018_816_MOESM6_ESM.pdf]

Results of sensitivity analyses with omission of one study at a time:

***** Sensitivity analyses were not performed at weeks 36 and 48 because there were just two studies.

--ALT level

Week 2

Influential analysis (Random effects model)

##

##	SMD 95%-CI	p-value	tau^2	I^2
## Omitting L. Peng, 2011	-0.4150 [-0.8401; 0.0101]	0.0557	0.1808	65.70%
**## Omitting L. Xu, 2014**	**-0.3224 [-0.6622; 0.0175]**	**0.0630**	**0.0721**	**41.30%**
## Omitting H. Lin, 2012	-0.4702 [-0.9515; 0.0111]	0.0555	0.2382	68.60%
## Omitting H. Luo, 2015	-0.5132 [-1.0288; 0.0024]	0.0511	0.2789	68.30%
## Omitting L. Tong, 2015	-0.5913 [-0.9924; -0.1902]	0.0039	0.1336	55.50%
## Omitting J. Yu, 2012	-0.4371 [-0.8940; 0.0198]	0.0608	0.2102	67.30%
## Omitting Y. Zhang, 2012	-0.5738 [-0.9924; -0.1552]	0.0072	0.1609	61.40%
##				
## Pooled estimate	-0.4740 [-0.8746; -0.0733]	0.0204	0.1757	62.60%

Week 12

Influential analysis (Random effects model)

##

##	SMD 95%-CI	p-value	tau^2	I^2
## Omitting Lin B-L,2017	-0.6135 [-1.1331; -0.0940]	0.0206	0.5873	85.90%
## Omitting Mehdi M, 2013	-0.6409 [-1.1266; -0.1551]	0.0097	0.5109	85.70%
## Omitting L. Peng, 2011	-0.5756 [-1.0594; -0.0917]	0.0197	0.5119	85.90%
## Omitting L. Xu, 2014	-0.4870 [-0.9426; -0.0315]	0.0361	0.4350	83.40%
## Omitting Y. Li,2015	-0.6756 [-1.1539; -0.1972]	0.0056	0.4830	83.90%
## Omitting H. Lin,2012	-0.5849 [-1.0874; -0.0825]	0.0225	0.5464	85.90%
## Omitting H. Luo,2015	-0.6327 [-1.1537; -0.1117]	0.0173	0.5898	85.50%
## Omitting S. Ouyang,2013	-0.6413 [-1.1476; -0.1349]	0.0131	0.5531	85.40%
## Omitting L. Tong,2015	-0.6659 [-1.1490; -0.1829]	0.0069	0.4980	84.90%
## Omitting Y. Zhang,2012	-0.5181 [-0.9911; -0.0452]	0.0318	0.4795	84.90%
**## Omitting L. Zhuang,2014**	**-0.3834 [-0.7204; -0.0465]**	**0.0257**	**0.1924**	**68.60%**
##				
## Pooled estimate	-0.5844 [-1.0398; -0.1290]	0.0119	0.4854	84.40%

Week 24

Influential analysis (Random effects model)

##

##	SMD 95%-CI	p-value	tau^2	I^2
## Omitting Lin B-L,2017	-1.0104 [-1.9871; -0.0338]	0.0426	1.6032	93.40%
## Omitting L. Peng, 2011	-0.9854 [-1.8783; -0.0924]	0.0306	1.3432	93.50%
## Omitting L. Xu, 2014	-0.7400 [-1.5690; 0.0891]	0.0802	1.1343	92.20%

## Omitting H. Lin,2012	-1.0125 [-1.9531; -0.0718]	0.0349	1.4823	93.40%
## Omitting S. Ouyang,2013	-1.0243 [-1.9921; -0.0565]	0.0380	1.5722	93.30%
## Omitting L. Tong,2015	-1.0898 [-1.9846; -0.1951]	0.0170	1.3309	92.90%
**## Omitting L. Zhuang,2014**	**-0.4228 [-0.8783; 0.0328]**	**0.0689**	**0.2726**	**74.50%**
## Omitting Suk KT,2017	-1.0151 [-1.9604; -0.0698]	0.0353	1.4974	93.40%
##				
## Pooled estimate	-0.9109 [-1.7205; -0.1013]	0.0274	1.2406	92.50%
