# Supplementary material for: A pooled analysis of mesenchymal stem cell-based therapy for liver disease
Source: Stem Cell Res Ther. 2018 Mar 21;9:72. doi: 10.1186/s13287-018-0816-2 (PMC5863358; doi:10.1186/s13287-018-0816-2)
Supplement: Supplementary file 7 — Visualized results of publication bias of ALT. (PDF 106 kb) [file 13287_2018_816_MOESM7_ESM.pdf]

Visualized results of publication bias of ALT

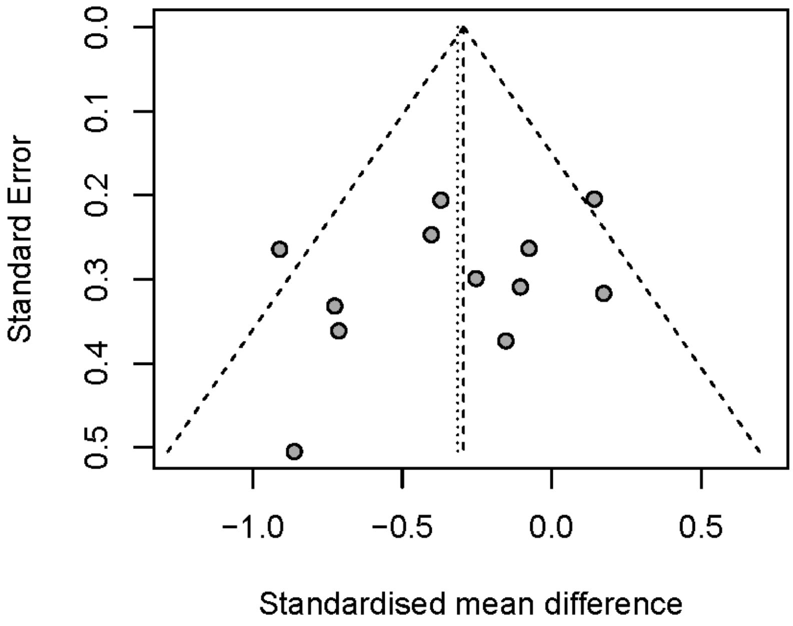

Figure S4. Bias assessment plot for ALT level at week 4
**p=0.29*

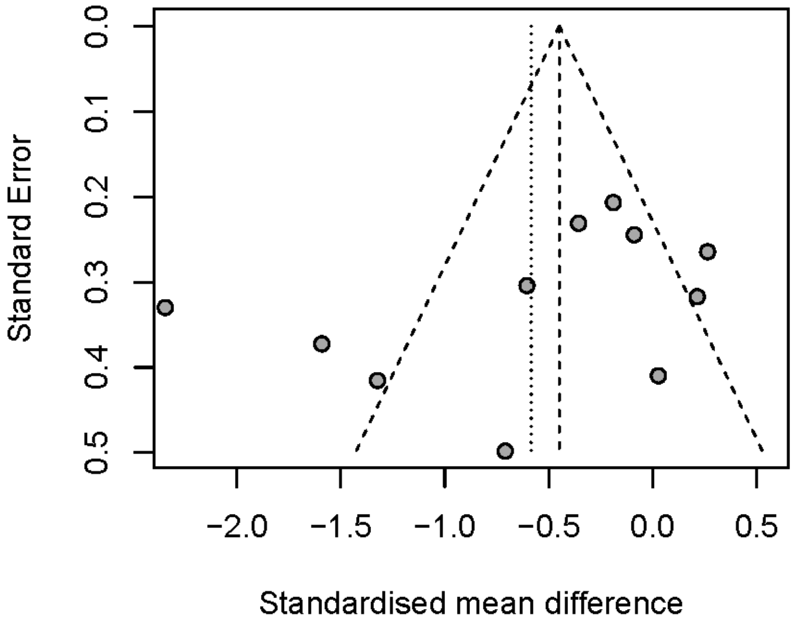

Figure S5. Bias assessment plot for ALT level at week 12
**p=0.19*
