# Supplementary material for: A pooled analysis of mesenchymal stem cell-based therapy for liver disease
Source: Stem Cell Res Ther. 2018 Mar 21;9:72. doi: 10.1186/s13287-018-0816-2 (PMC5863358; doi:10.1186/s13287-018-0816-2)
Supplement: Supplementary file 8 — Results of sensitivity analyses: TBiL levels. (PDF 54 kb) [file 13287_2018_816_MOESM8_ESM.pdf]

Results of sensitivity analyses with omission of one study at a time:

***** Sensitivity analyses were not performed at weeks 36 and 48 because there were just two studies.

--TBiL level

Week 2

Influential analysis (Random effects model)

##

##	SMD 95%-CI	p-value	tau^2	I^2
## Omitting L. Peng, 2011	-0.4290 [-0.8296; -0.0285]	0.0358	0.2674	72.80%
## Omitting Salama H,2014	-0.3867 [-0.7948; 0.0213]	0.0632	0.2704	71.70%
**## Omitting L. Xu, 2014**	**-0.2702 [-0.5681; 0.0276]**	**0.0753**	**0.0970**	**48.40%**
## Omitting Z. Zhang, 2012	-0.4372 [-0.8541; -0.0204]	0.0398	0.2866	72.70%
## Omitting H. Lin,2012	-0.4542 [-0.8702; -0.0381]	0.0324	0.2835	72.20%
## Omitting B. Liu, 2013	-0.3119 [-0.6622; 0.0384]	0.0810	0.1748	63.00%
## Omitting H. Luo,2015	-0.4601 [-0.8923; -0.0279]	0.0369	0.3082	71.30%
## Omitting L. Tong,2015	-0.4933 [-0.8819; -0.1048]	0.0128	0.2340	68.60%
## Omitting J. Yu,2012	-0.4264 [-0.8390; -0.0137]	0.0428	0.2812	72.80%
## Omitting Y. Zhang,2012	-0.4858 [-0.8756; -0.0959]	0.0146	0.2406	69.90%
##				
## Pooled estimate	-0.4151 [-0.7866; -0.0437]	0.0285	0.2414	69.40%

Week 4

Influential analysis (Random effects model)

##

##	SMD 95%-CI	p-value	tau^2	I^2
## Omitting Lin B-L,2017	-0.4457 [-0.7567; -0.1348]	0.0050	0.2398	72.70%
## Omitting L. Peng, 2011	-0.3968 [-0.6868; -0.1068]	0.0073	0.2079	72.30%
## Omitting Salama H,2014	-0.3891 [-0.6815; -0.0966]	0.0091	0.2065	71.50%
**## Omitting L. Xu, 2014**	**-0.2622 [-0.4694; -0.0549]**	**0.0132**	**0.0657**	**45.00%**
## Omitting Z. Zhang, 2012	-0.4329 [-0.7335; -0.1323]	0.0048	0.2228	72.90%
## Omitting H. He,2015	-0.4566 [-0.7633; -0.1499]	0.0035	0.2305	71.90%
## Omitting Y. Li,2015	-0.4490 [-0.7513; -0.1467]	0.0036	0.2239	72.50%
## Omitting H. Lin,2012	-0.4134 [-0.6945; -0.1324]	0.0039	0.2033	70.90%
## Omitting H. Luo,2015	-0.4671 [-0.7623; -0.1719]	0.0019	0.2065	69.70%
## Omitting S. Ouyang,2013	-0.4169 [-0.7215; -0.1124]	0.0073	0.2280	72.60%
## Omitting L. Tong,2015	-0.4572 [-0.7519; -0.1625]	0.0024	0.2106	71.80%
## Omitting Q. Wang,2013	-0.4051 [-0.6943; -0.1159]	0.0060	0.2098	72.70%
## Omitting J. Yu,2012	-0.4204 [-0.7187; -0.1220]	0.0058	0.2197	72.90%
## Omitting Y. Zhang,2012	-0.3298 [-0.5901; -0.0696]	0.0130	0.1494	65.10%
## Omitting M. Zhu,2013	-0.4354 [-0.7363; -0.1345]	0.0046	0.2231	72.90%
## Omitting L. Zhuang,2014	-0.4428 [-0.7479; -0.1377]	0.0044	0.2292	72.80%
##				
## Pooled estimate	-0.4134 [-0.6945; -0.1324]	0.0039	0.2033	70.90%

Week 8

Influential analysis (Random effects model)

##	SMD 95%-CI	p-value	tau^2	I^2
## Omitting Lin B-L,2017	-0.7647 [-1.4391; -0.0903]	0.0263	0.6899	88.10%
## Omitting L. Xu, 2014	-0.4871 [-1.0734; 0.0992]	0.1035	0.5066	86.40%
## Omitting Z. Zhang, 2012	-0.7076 [-1.3664; -0.0488]	0.0353	0.6611	88.90%
## Omitting H. He,2015	-0.6986 [-1.4065; 0.0093]	0.0531	0.7705	88.80%
## Omitting Y. Li,2015	-0.7389 [-1.4106; -0.0671]	0.0311	0.6867	88.70%
## Omitting H. Lin,2012	-0.7589 [-1.4050; -0.1128]	0.0213	0.6301	88.30%
**## Omitting Y. Zhang,2012**	**-0.2463 [-0.5818; 0.0892]**	**0.1502**	**0.1267**	**63.30%**
## Omitting M. Zhu,2013	-0.7069 [-1.3679; -0.0460]	0.0361	0.6656	88.90%
##				
## Pooled estimate	-0.6165 [-1.1920; -0.0411]	0.0357	0.5665	87.10%

Week 12

Influential analysis (Random effects model)

##	SMD 95%-CI	p-value	tau^2	I^2
## Omitting D Zhang,2016	-0.6193 [-0.9214; -0.3172]	< 0.0001	0.2494	74.10%
## Omitting Lin B-L,2017	-0.6470 [-0.9357; -0.3583]	< 0.0001	0.2181	70.90%
## Omitting L. Peng, 2011	-0.5894 [-0.8802; -0.2985]	< 0.0001	0.2333	74.00%
## Omitting Salama H,2014	-0.5818 [-0.8763; -0.2874]	0.0001	0.2352	73.60%
## Omitting L. Xu, 2014	-0.5066 [-0.7612; -0.2520]	< 0.0001	0.1554	65.20%
## Omitting Z. Zhang, 2012	-0.6089 [-0.9074; -0.3104]	< 0.0001	0.2439	74.20%
## Omitting H. He,2015	-0.6287 [-0.9351; -0.3224]	< 0.0001	0.2569	73.60%
## Omitting Y. Li,2015	-0.6323 [-0.9302; -0.3344]	< 0.0001	0.2400	73.40%
## Omitting H. Lin,2012	-0.6342 [-0.9289; -0.3395]	< 0.0001	0.2343	73.30%
## Omitting B. Liu, 2013	-0.6305 [-0.9216; -0.3395]	< 0.0001	0.2304	73.50%
## Omitting H. Luo,2015	-0.5892 [-0.8925; -0.2859]	0.0001	0.2503	73.40%
## Omitting S. Ouyang,2013	-0.6198 [-0.9231; -0.3164]	< 0.0001	0.2517	74.10%
## Omitting L. Tong,2015	-0.6416 [-0.9314; -0.3519]	< 0.0001	0.2242	72.50%
**## Omitting Y. Zhang,2012**	**-0.4964 [-0.7363; -0.2565]**	**< 0.0001**	**0.1317**	**61.70%**
## Omitting L. Zhuang,2014	-0.5694 [-0.8619; -0.2769]	0.0001	0.2281	72.40%
## Omitting El-Ansary M,2012	-0.5710 [-0.8592; -0.2828]	0.0001	0.2256	73.30%
##				
## Pooled estimate	-0.5970 [-0.8774; -0.3165]	< 0.0001	0.2242	72.40%

Week 24

Influential analysis (Random effects model)

##	SMD 95%-CI	p-value	tau^2	I^2
## Omitting Lin B-L,2017	-0.5147 [-0.9322; -0.0973]	0.0157	0.3380	76.50%

## Omitting L. Peng, 2011	-0.4421 [-0.8696; -0.0145]	0.0427	0.3757	80.40%
## Omitting Salama H,2014	-0.3694 [-0.7840; 0.0452]	0.0808	0.3385	78.00%
## Omitting Suk KT,2017	-0.4844 [-0.9237; -0.0451]	0.0307	0.3890	79.70%
**## Omitting L. Xu, 2014**	**-0.2554 [-0.5457; 0.0348]**	**0.0845**	**0.1205**	**56.60%**
## Omitting Z. Zhang, 2012	-0.4484 [-0.8908; -0.0060]	0.0470	0.3981	80.40%
## Omitting H. Lin,2012	-0.5074 [-0.9311; -0.0837]	0.0189	0.3553	78.40%
## Omitting S. Ouyang,2013	-0.4433 [-0.8983; 0.0118]	0.0562	0.4229	80.40%
## Omitting L. Tong,2015	-0.4859 [-0.9208; -0.0510]	0.0286	0.3811	79.70%
## Omitting L. Zhuang,2014	-0.3917 [-0.8235; 0.0401]	0.0754	0.3707	78.60%
## Omitting El-Ansary M,2012	-0.4526 [-0.8851; -0.0200]	0.0403	0.3821	80.40%
##				
## Pooled estimate	-0.4344 [-0.8350; -0.0339]	0.0335	0.3495	78.30%
