# Supplementary material for: A pooled analysis of mesenchymal stem cell-based therapy for liver disease
Source: Stem Cell Res Ther. 2018 Mar 21;9:72. doi: 10.1186/s13287-018-0816-2 (PMC5863358; doi:10.1186/s13287-018-0816-2)
Supplement: Supplementary file 9 — Visualized results of publication bias of TBiL. (PDF 133 kb) [file 13287_2018_816_MOESM9_ESM.pdf]

Visualized results of publication bias of TBiL

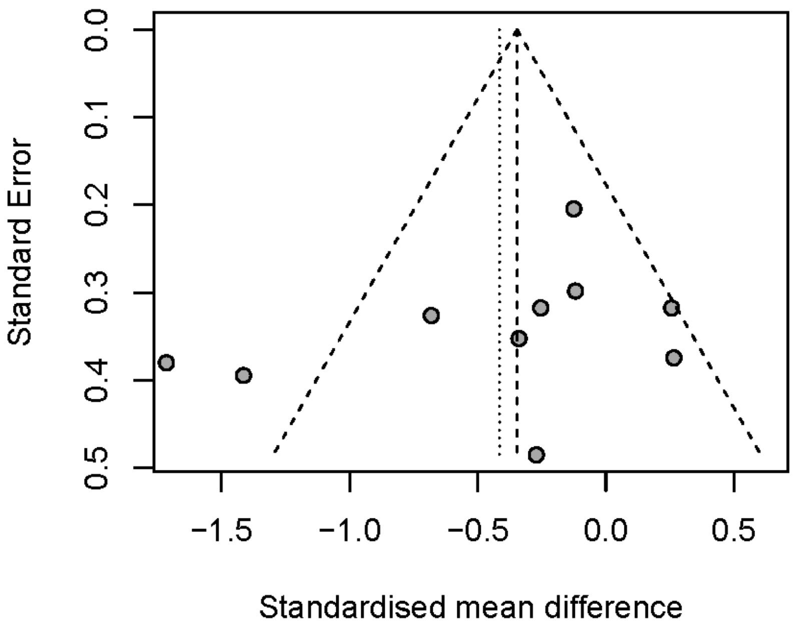

Figure S6. Bias assessment plot for TBiL level at week 2
*p=0.28

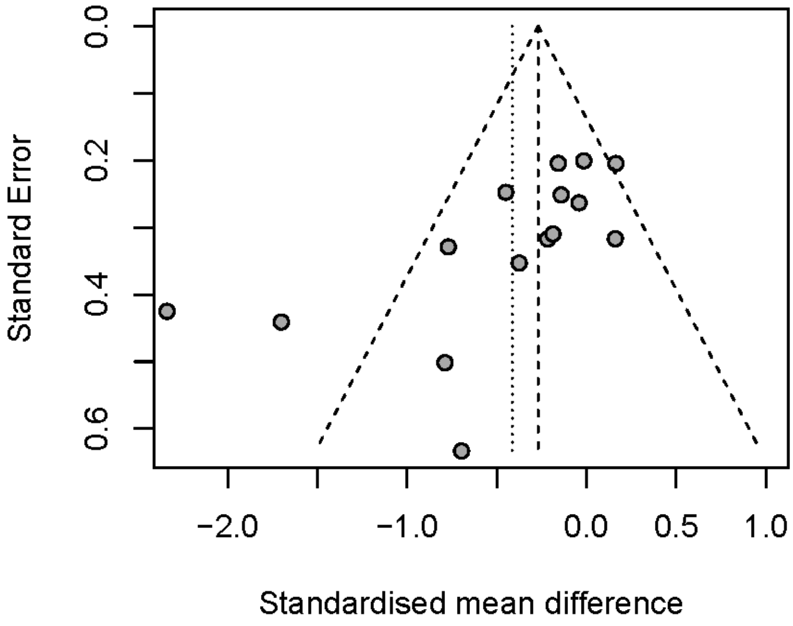

Figure S7. Bias assessment plot for TBiL level at week 4
**p<0.01*

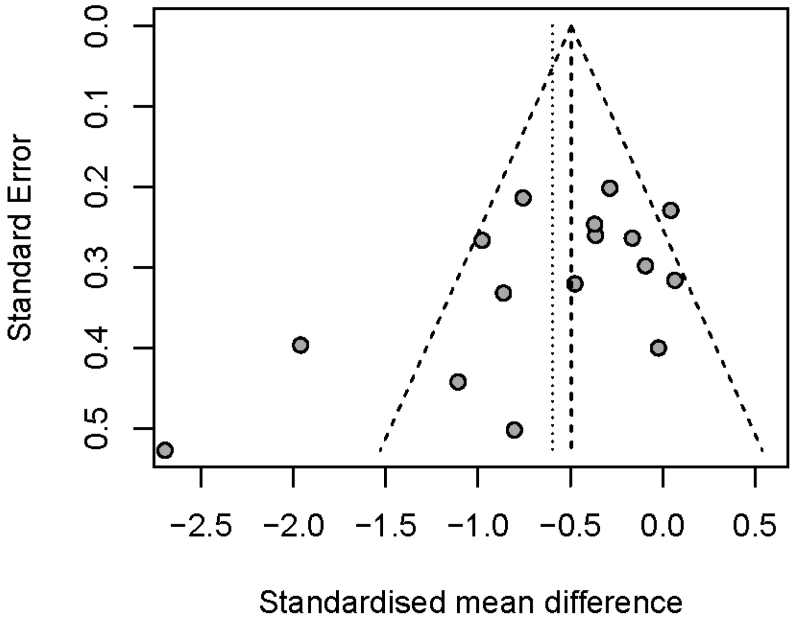

Figure S8. Bias assessment plot for TBiL level at week 12
**p=0.04*

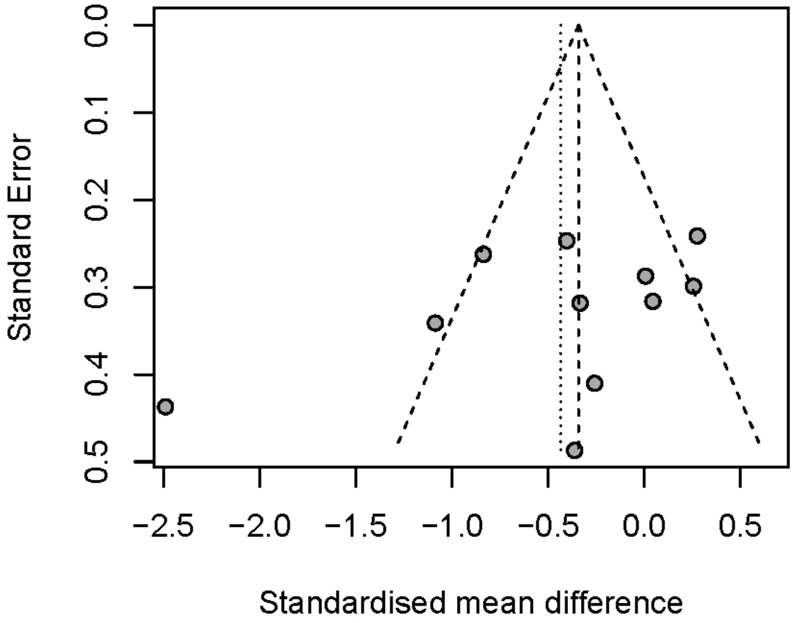

Figure S9. Bias assessment plot for TBiL level at week 24
**p=0.17*
