# Supplementary material for: A pooled analysis of mesenchymal stem cell-based therapy for liver disease
Source: Stem Cell Res Ther. 2018 Mar 21;9:72. doi: 10.1186/s13287-018-0816-2 (PMC5863358; doi:10.1186/s13287-018-0816-2)
Supplement: Supplementary file 10 — Results of symmetrical contour-enhanced funnel plots combined with trim and fill analysis of TBiL. (PDF 167 kb) [file 13287_2018_816_MOESM10_ESM.pdf]

Results of symmetrical contour-enhanced funnel plots combined with trim and fill analysis of TBiL:

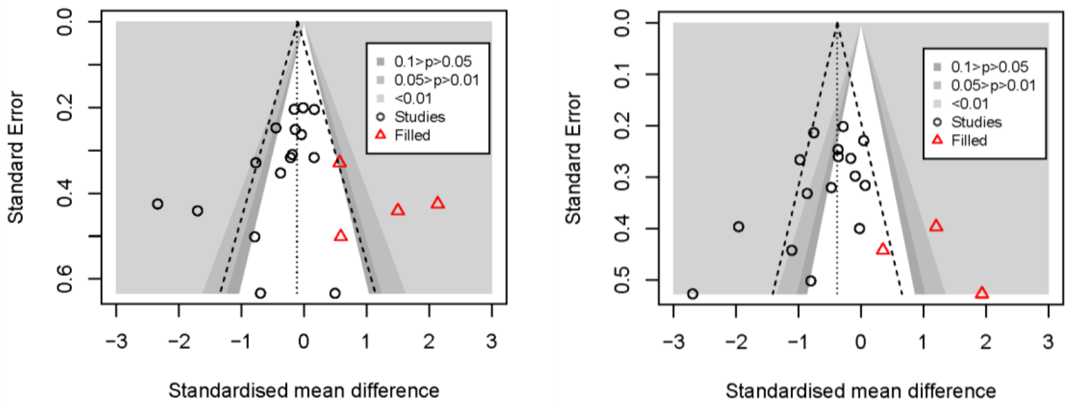

Figure S11 Symmetrical contour-enhanced funnel plots for TBiL at weeks 4 and 12

At week 4, four hypothetical studies were filled: three plotted in the area of statistical significant and one in the area of statistical nonsignificant; at week 12, three hypothetical studies were filled: two plotted in the area of statistical significant and one in the area of statistical nonsignificant, indicating that the asymmetry was partly caused by publication bias.
